# Optical coherence tomography angiography of superficial retinal vessel density and foveal avascular zone in myopic children

**DOI:** 10.1371/journal.pone.0219785

**Published:** 2019-07-18

**Authors:** Joanna Gołębiewska, Karolina Biała-Gosek, Agnieszka Czeszyk, Wojciech Hautz

**Affiliations:** Department of Ophthalmology, The Children's Memorial Health Institute, Warsaw, Poland; Nicolaus Copernicus University, POLAND

## Abstract

**Purpose:**

To assess the superficial retinal vessel density (SRVD) and foveal avascular zone (FAZ) in myopic children using optical coherence tomography angiography (OCTA).

**Methods:**

174 eyes of 89 subjects with myopia and 101 eyes of 54 age-matched, emmetropic volunteers (control group) were enrolled in this study. The mean age of the subjects and controls was 13.9 (SD ± 2.3) and 13.1 (SD ± 2.4), respectively. Myopia was defined as spherical equivalent <– 1.0 diopter. Emmetropic subjects were defined as having spherical equivalent from + 0.5 to − 0.5 diopter. The mean axial length (AL) in myopic patients was 24.58 mm (SD ± 1.22) and 22.88 mm (SD ± 0.65) in the controls. Every patient underwent a complete ophthalmological examination and OCTA, using AngioVue (Optovue). The FAZ area and superficial retinal vessel density, including whole SRVD, fovea SRVD and parafovea SRVD, were analyzed. Foveal thickness (FT) and parafoveal thickness (PFT) were also taken into consideration.

**Results:**

Whole SRVD, parafovea SRVD and PFT were significantly higher in controls than in the myopic subjects (*p* < 0.001, *p* = 0.007, p < 0.01, respectively). The FAZ area was significantly larger in the myopic group compared to the controls (p = 0.010). Fovea SRVD and FT did not differ significantly between the groups (*p* = 0.740, *p* = 0.795 respectively). In overall subjects we found significant correlation between axial length and all the investigative parameters: age, FAZ area, whole SRVD, parafovea SRVD, fovea SRVD, PFT, FT (*p* < 0.001, *p* = 0.014, *p* = 0.008, p < 0.005, *p* = 0.014, p = 0.010, p = 0.024, respectively). Analyzing only myopic group we confirmed that AL was significantly correlated with age, whole SRVD and parafovea SRVD (*p* < 0.001, *p* = 0.014, *p* = 0.009, respectively). Similarly, in this group the spherical equivalent also correlated with age, whole SRVD and parafovea SRVD (*p* < 0.001, *p* = 0.007, *p* = 0.005, respectively). Such correlations were not confirmed in the non–myopic group.

**Conclusions:**

Our results suggest that superficial retinal vessel density is decreased and FAZ area is enlarged in the entire group of the myopic children compared to emmetropic subjects. Longitudinal observation of these young patients is needed to determine the relevance of the microvascular alterations in future.

## Introduction

Myopia, defined as refractive error due to excessive elongation of the eye, is a rising problem in pediatric population around the world. [1,2] The retinal complications of myopia, which threaten the vision, include retinal detachment and myopic maculopathy. Several authors emphasize the role of optical coherence tomography (OCT) in non-invasive, detailed evaluation of pediatric retina, which is very important not only in retinal disorders but also in understanding the normal eye growth. [3–6] Optical coherence tomography angiography (OCTA) is a new, non-invasive tool, involving the detection of intravascular erythrocyte movement. [7] OCTA enables reproducible, quantitative assessment of the macular microcirculation in the macula and may be used in diagnosing different retinal diseases, such as diabetic retinopathy, central serous chorioretinopathy and age-related macular degeneration. [8–10] OCTA provides three-dimensional maps of the macular perfusion and seems to be a promising method in the detection of early microcirculation disorders. To the best of our knowledge there are few reports on retinal perfusion in myopic adults using this method but no previous reports on OCTA findings in myopic children. [11–14]

The aim of the study was to assess the superficial retinal vessel density (SRVD) and foveal avascular zone area (FAZ) in myopic children using OCT angiography and to compare potential pathologic changes in this population to emmetropic age-matched controls.

## Material and methods

This observational, cross–sectional study was conducted in The Children's Memorial Health Institute in Warsaw from January 2017 to September 2017 and enrolled patients recruited from routine visits to the ophthalmology outpatient department, who met inclusion criteria. This study was approved by the Bioethics Committee of The Children's Memorial Health Institute in Warsaw and followed the tenets of the Declaration of Helsinki. After explanation of the nature and possible consequences of the study, a written informed consent was obtained from the patient’s legal guardian and from patients above 16 years of age. The study eyes were divided into two groups based on mean spherical equivalent (MSE): myopic (MSE <– 1.0 diopters (D)) and non–myopic (MSE 0.50 D to − 0.50 D). MSE was measured by cycloplegic autorefraction after administration of 1% tropicamide drops 3 times every 5 minutes (Nidek, Gamagori, Japan).

Exclusion criteria in both groups were the history of prematurity, other concomitant retinal pathologies, such as hereditary retinal dystrophies, vitreoretinal diseases, the history of ocular trauma, neurological disorders, glaucoma, amblyopia, previous retinal laser treatment and lack of cooperation. Eyes with poor quality scans were also excluded. Every patient underwent a complete ophthalmic examination, including best-corrected visual acuity (BVCA) using Snellen’s chart, slit-lamp biomicroscopy, dilated fundus examination and color fundus photography. Axial length (AL) was measured using the OcuScan (Alcon, Fort Worth, US). Three separate measurements were performed in total, and the average value was recorded.

**Fig 1:**
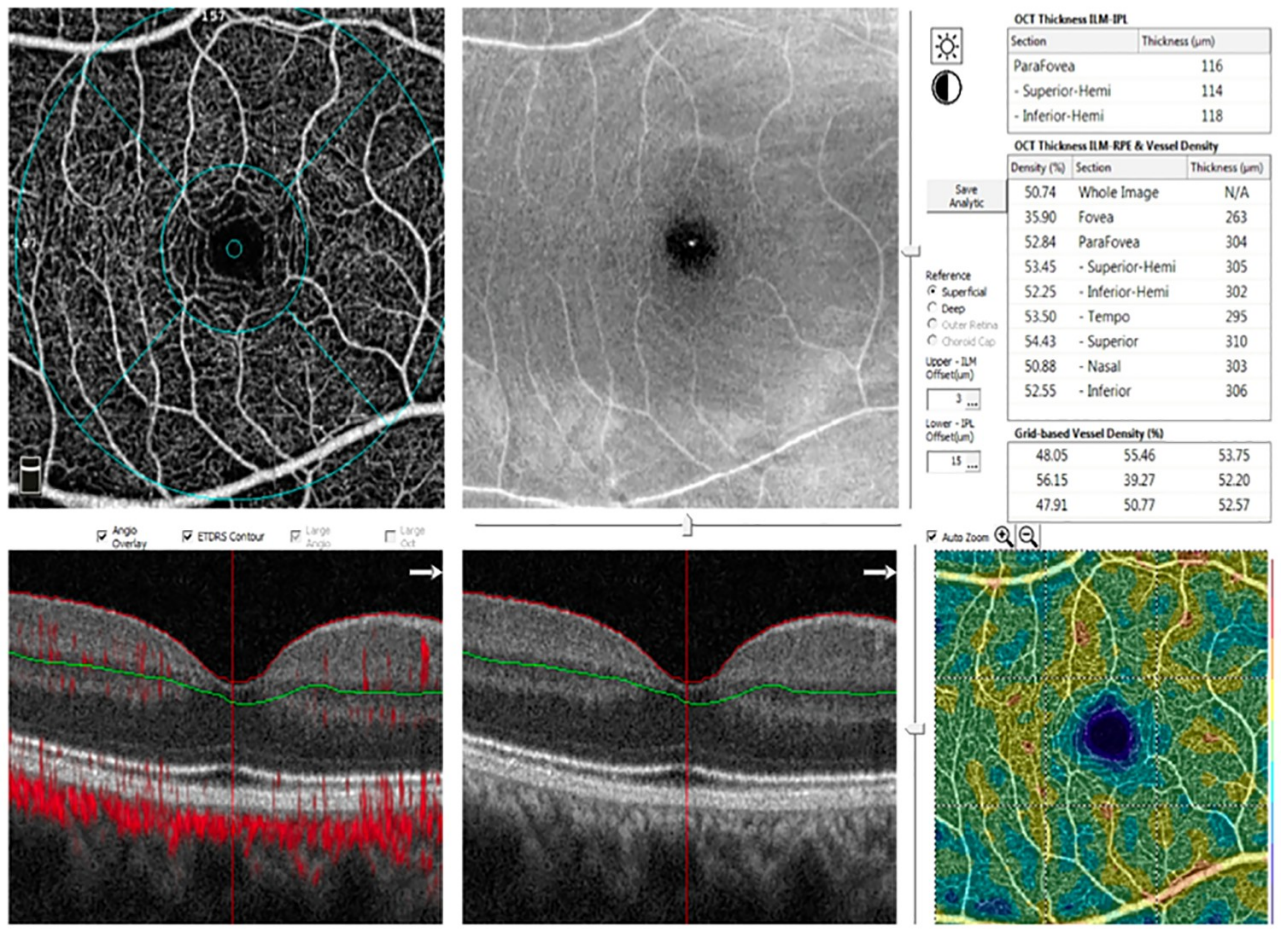
Representative OCTA vessel density report of myopic eye. Figure panel shows: the image of the macular vessels, separately calculated in five regions (fovea, temporal, superior, nasal and inferior) based on the ETDRS contour, OCT en face image, B-scans, and outcomes of quantitative analysis by the software.

OCTA was performed using a commercially available RTVue XR Avanti with AngioVue (Optovue, Fremont, CA, USA) with 3 mm x 3 mm images of the macula, centered on the foveola. Each OCTA en face image contains 304 x 304 pixels created from the intersection of the 304 vertical and the 304 horizontal B-scans. AngioVue automatically segments the area into four layers, including superficial capillary plexus layer (SP), deep capillary plexus layer (DP), outer retina layer and choriocapillaries. The SP en face image was segmented with an inner boundary at 3 μm beneath the internal limiting membrane and an outer boundary set at 15 μm beneath the inner plexiform layer, whereas the deep capillary plexus en face image was segmented with an inner boundary 15 μm beneath the inner plexiform layer and an outer boundary at 70 μm beneath the inner plexiform layer. Integrated automated algorithms provided by the machines software were used to quantify FAZ area (mm^2^) and macular vascular density (%) in superficial plexus. The whole superficial retinal vessel density, fovea SRVD, parafovea SRVD were taken into analysis. The parafoveal area as defined by the 3 mm partial ETDRS grid from the AngioVue software is the area comprised between the 1–3 mm concentric ring centered of the fovea. The parafoveal area is then further divided into 4 sectors for Quadrant analysis (temporal (T), superior (S), nasal (N) and inferior (I)) or 2 Hemispheres (Superior (S_Hemi) and Inferior (I_Hemi), divided by horizontal line through the foveal center. Fig 1 To avoid inaccuracy in FAZ measurements due to ocular magnification we used Matlab script (Mathworks, Natick, MA), previously described by Linderman at al. [15] The area of the FAZ was calculated as follows: A_corrected_ = A _nominal_ (ALs/Alm)^2^, where *ALs*—axial length of the subject in mm, *Alm*—axial length assumed for the model eye (23.95 mm).

To avoid the impact of axial length variation on the vessel density measurement we corrected the magnification error using the Littman and the modified Bennett formulae, as Sampson described. [16] Thus, the magnification factor of the image should be corrected by: D_t_^2^/D_m_^2^ = 0.002066 (AL– 1.82)^2^, where D_m_ is a empirically measured fundus diameter, D_t_ = 23.82 mm (true fundus diameter according to the Bennett formulae), and 1.82 is a constant related to the distance between the corneal apex and the second principal plane and AL is the axial length.

Foveal thickness (FT) (μm) and parafoveal thickness (PFT) (μm) data were obtained from retinal maps, using the same device. Three scans for each eye were captured, then the best one in quality (with a signal strength index > 6) was considered for analysis. Trained OCTA readers (JG, KBG) reviewed all images independently to ensure correct segmentation and identify poor quality scans, with motion artifacts or blurred images, where data were insufficient for proper analysis. The data collected from both eyes of the studied patients were taken into analysis.

### Statistical analysis

The variables were expressed as means, standard deviations, 95% confidence intervals, and ranges. The one-way multifactor analysis of variance (ANOVA) was used to determine the differences between patients and controls, if the assumptions of normality of distribution and homogeneity of variances were met, or generalized linear models with robust standard errors, when said assumptions were violated. Linear relationships between selected quantitative variables were assessed using the Pearson product-moment correlation coefficient. All the statistical models fitted were corrected for study participants’ age and gender when applicable, and incorporated intra-subject standard errors (two eyes of one patients).

A level of *p* < 0.05 was considered statistically significant for all comparisons. All the statistical computations were carried out using Stata/Special Edition, release 14.2 (StataCorp LP, College Station, Texas, USA).

## Results

**Fig 2:**
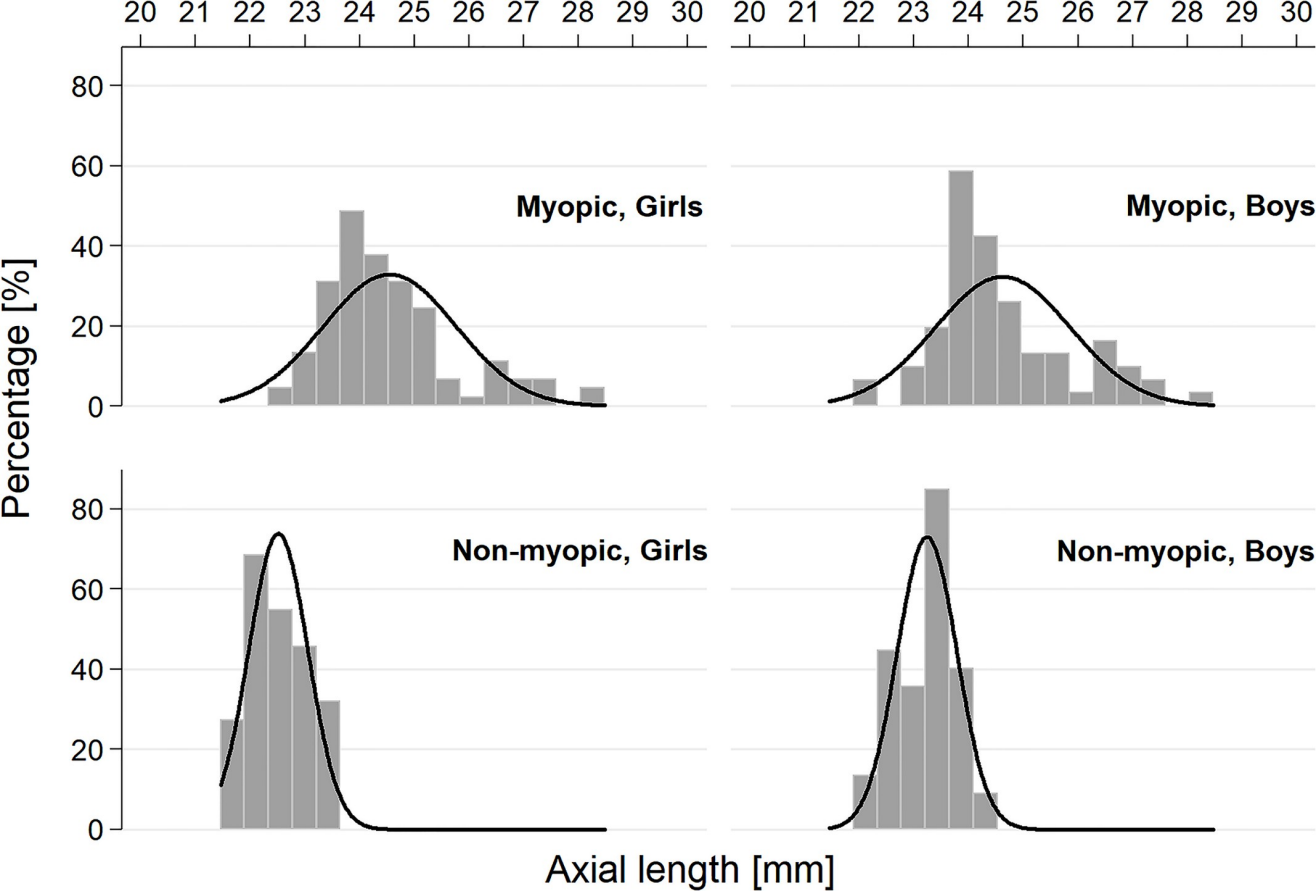
Histogram depicting the distribution of axial length in the studied patients by presence of myopia and gender.

Ninety-six consecutive children with myopia and sixty emmetropic children were recruited to this study. After exclusion of eyes with poor quality OCTA images, 89 myopic children (174 eyes) were taken to the final analysis. 54 emmetropic children (101 eyes) constituted their age-matched control group. The mean age of the subjects and controls was 13.9 (SD ± 2.3) and 13.1 (SD ± 2.4) years, respectively. The mean AL in myopic patients was 24.58 mm (SD ± 1.22) and 22.88 mm (SD ± 0.65) in controls. Fig 2 The in-depth descriptive characteristics of the entire cohort are shown in Table 1.

**Table 1:** Characteristics of the studied patients.

Variable	Statistical parameter			
	*M*	*SD*	*95% CI*	*Min*.—*max*.
**Age (years)**				
**Myopic**	13.9	2.3	13.6–14.3	9–17
**Non-myopic**	13.1	2.4	12.7–13.6	9–18
**Spherical equivalent (D)** (myopic group only)				
**Right eye**	–3.61	2.48	–3.08 to –4.14	–0.25 to –11.75
**Left eye**	–3.30	2.44	–2.76 to –3.83	–0.25 to –12.00
**Axial length (mm)**				
**Myopic**	24.58	1.22	24.40–24.76	22.04–28.48
**Non-myopic**	22.88	0.65	22.75–23.01	21.46–24.14

(M—mean; SD—standard deviation; CI—confidence interval. Multivariate analyses were performed, taking into account the patients’ age and sex)

Whole SRVD, parafovea SRVD and PFT were significantly higher in controls than in the myopic subjects (*p* < 0.001, *p* = 0.007, p < 0.01, respectively). The FAZ area was significantly larger in the myopic group compared to the controls (p = 0.010). The fovea SRVD and FT did not differ significantly between the groups (*p* = 0.740, *p* = 0.795 respectively). Descriptive measures for investigated ophthalmic parameters in both study groups are summarized in Table 2.

**Table 2:** Descriptive statistics for selected features of the fovea and the parafovea in the studied patients by presence of myopia.

Variable	Myopic				Non-myopic				* Level of statistical significance
	*M*	*SD*	*95% CI*	*Min*.—*max*.	*M*	*SD*	*95% CI*	*Min*.—*max*.	
**Axial length corrected foveal avascular zone, FAZ (mm**^**2**^**)**	0.258	0.091	0.245–0.272	0.029–0.563	0.224	0.076	0.209–0.239	0.004–0.446	***p* = .010**
**Axial length corrected whole superficial vessel density, wsVD (%)**	48.03	4.04	47.24–48.83	37.04–57.76	54.54	6.04	53.64–55.45	39.93–77.67	***p* < .001**
**Foveal superficial vessel density, fsVD (%)**	31.64	4.82	30.92–32.36	19.75–44.09	31.63	4.40	30.76–32.47	22.00–40.22	*p* = 0.740
**Parafoveal superficial vessel density, psVD (%)**	53.18	3.33	52.68–53.67	41.94–62.08	54.51	3.08	53.90–55.12	42.64–59.06	***p* = .007**
**Foveal thickness, FT (μm)**	252.09	18.56	249.31–254.86	204–292	251.11	19.93	247.17–255.04	213–316	*p* = .795
**Parafoveal thickness, PFT (μm)**	311.80	18.90	308.97–314.63	195–356	321.22	13.55	318.54–323.89	284–350	***p* < .001**

(M—mean; SD—standard deviation; CI—confidence interval. Multivariate analyses were performed, taking into account the patients’ age and sex)

In overall subjects we found significant correlation between axial length and all the investigative parameters: age, FAZ area, whole SRVD, parafovea SRVD, fovea SRVD, PFT, FT (*p* < 0.001, *p* = 0.014, *p* = 0.008, p < 0.005, *p* = 0.014, p = 0.010, p = 0.024, respectively). Analyzing separately myopic group we confirmed that AL was significantly correlated with age, whole SRVD and parafovea SRVD (*p* < 0.001, *p* = 0.014, *p* = 0.009, respectively). Similarly, in this group the spherical equivalent also correlated with age, whole SRVD and parafovea SRVD (*p* < 0.001, *p* = 0.007, *p* = 0.005, respectively). Such correlations were not found in the non–myopic group. Table 3

**Table 3:** Pearson’s correlation coefficients and p-values for the axial length and spherical equivalent *versus* selected traits in the studied patients by presence of myopia.

Study group	Myopic				Non-myopic		Overall	
Dependent variable	Axial length		Spherical equivalent		Axial length		Axial length	
Statistical parameter	*r* *	*p* ^†^	*r*	*p*	*r*	*p*	*r*	*p*
**Age**	0.34	< 0.001	0.34	< 0.001	–0.03	= 0.700	0.31	< 0.001
**FAZ**	–0.14	= 0.145	–0.06	= 0.502	–0.16	= 0.163	–0.19	= 0.014
**Whole SRVD**	–0.22	= 0.014	–0.24	= 0.007	0.14	= 0.231	–0.21	= 0.008
**Fovea SRVD**	0.15	= 0.109	0.08	= 0.366	0.15	= 0.190	0.19	= 0.014
**Parafovea SRVD**	–0.23	= 0.009	–0.24	= 0.005	0.13	= 0.251	–0.22	= 0.005
**FT**	0.13	= 0.169	0.07	= 0.447	0.17	= 0.154	0.18	= 0.024
**PFT**	–0.18	= 0.126	–0.17	= 0.158	0.08	= 0.370	–0.20	= 0.010

* Pearson product-moment correlation coefficient;

^†^ level of statistical significance.

Multivariate analyses were carried out, hence all the correlation coefficients and p-values shown were controlled for the studied patients’ age and sex, except the age that was corrected for sex only)

## Discussion

In this study superficial retinal vessel density and FAZ area were measured in myopic and emmetropic children using non–invasive OCT angiography. Although the exact etiology of myopia remains unclear, it typically manifests itself and develops in childhood and adolescence, from about the age of 7–8 and is known to be associated with excessive elongation of the axial length of the eye. A number of recent studies using the SD-OCT proved the impact of myopia and refractive error upon retinal thickness and morphology, nerve fibre layer thickness, ganglion cell complex and choroidal thickness. [17–19] In agreement with Read and al. we found decreased parafoveal thickness in myopic children, which may confirm redistribution of retinal thickness related to the increased axial length of myopic eyes. [6] The authors report that the axial stretching of the eye may provoke the development of various retinal and choroidal complications, mainly in high myopia. The range of the complications includes decreased blood flow and the narrowing of retinal vessels. [11–14,20,21] Similarly, decreased choriocapillaris density and diameter are reported both in animal models of myopia and in human subjects. [22–24] The exact mechanism of decreased perfusion in myopic eyes remains unknown, some authors indicate that axial stretching of the eye may be partially responsible for the altered vascular network and those changes may be related to the pathogenesis of pathological myopia. [21–23] To the best of our knowledge all previous studies based on OCT angiography findings describe reduced perfusion in adults with different stages of myopia. Hence, we decided to assess vessel density in myopic children to find out if similar pathologies also concern them. Fan at al. evaluated vascular density in macula and optic disc region in eyes with different refractive statuses to determine factors associated with the vascular density. They found that longer AL is associated with decreased superficial and deep vascular density. [11] Our results confirmed this correlation in superficial retinal plexus in children. Mo and al. measured macular, choriocapillaris and radial peripapillary flow density (RPC) in the eyes with emmetropia, high myopia and pathological myopia. The authors found significant decrease of macular and RPC flow only in the group with pathological myopia and confirmed negative correlation between flow density and AL. In the present study analyzing overall subjects we found significant correlation between AL and all the investigative parameters. Analyzing separately both groups we confirmed that decreased superficial vascular density in macular area was strongly associated with longer AL only in myopic patients. It may indicate that elongation of the eye in myopia is an important parameter affecting vascular density. In agreement with Mo and al. our results proved negative correlation between SRVD and AL and refractive error. [14] Similarly, Yang at al. showed that ocular blood flow was negatively related to AL. [24] Linderman and Sampson focused on inaccuracy in FAZ and vessel density measurements due to ocular magnification. [15,16] To avoid the impact of axial length variation on the vessel density and FAZ measurement we corrected the ocular magnification error using the described formulas. The main limitation of the study is poor representativeness of the sample—it is single—centre study with monoracial background- all subjects were Caucasian, and the lack of differences in this clinical population may not reflect the entire cohort of myopic children across the world. The mechanism of decreased macular vascular density in children with myopia needs further research.

## Conclusions

Our results suggest that superficial retinal vessel density is decreased and FAZ area is enlarged in the entire group of the myopic children compared to emmetropic subjects. Longitudinal observation of these young patients is needed to determine the relevance of the microvascular alterations in future.

## Supporting information

S1 File Database. (XLSX)

## References

[1] Rudnicka AR, Kaptenakis VV, Wathern AK, Logan NS, Gilmartin B, Whincup PH et al Global variations and time trends in the prevalence of childhood myopia, a systematic review and quantitative metaanalysis: implications for aetiology and early prevention. Br J Ophthalmol 2016; 100: 882–890. 10.1136/bjophthalmol-2015-307724 26802174 PMC4941141

[2] Holden BA, Fricke TR, Wilson DA, Jong M, Naidoo KS, Sankaridurg P et al Global prevalence of myopia and high myopia and temporal trends from 2000 through 2050. Ophthalmology 2016; 123:1036–1042. 10.1016/j.ophtha.2016.01.006 26875007

[3] Lee H, Proudlock FA, Gottlob I. Pediatric optical coherence tomography in clinical practice- recent progress. Invest Ophthalmol Vis Sci 2016; 57: OCT69–OCT79. 10.1167/iovs.15-18825 27409508

[4] Turk A, Ceylan OM, Arici C, Keskin S, Erdurman C, Durukan AH et al Evaluation of the nerve fiber layer and macula in the eyes of healthy children using spectral domain optical coherence tomography. Am J Ophthalmol 2012; 153: 552–559. 10.1016/j.ajo.2011.08.026 22019223

[5] Li T, Zhou X, Wang Z, Zhu J, Shen W, Jiang B et al Assessment of retinal and choroidal measurements in Chinese school-age children with Cirrus-HD optical coherence tomography. PLoS One 2016; 11: e0158948 10.1371/journal.pone.0158948 27391015 PMC4938617

[6] Read SA, Alonso-Caneiro D, Vincent SJ. Longitudinal changes in macular retinal layer thickness in pediatric populations: Myopic vs non-myopic eyes. PLoS One 2017; 12(6): e0180462 10.1371/journal.pone.0180462 28662138 PMC5491256

[7] Jia Y, Tan O, Tokayer J, Potsaid B, Wang Y, Liu JJ et al Split-spectrum amplitude decorrelation angiography with optical coherence tomography. Opt Express 2012; 20:4710–4725. 10.1364/OE.20.004710 22418228 PMC3381646

[8] Durbin M, An L, Shemonski ND, Soares M, Santes T, Lopes M et al Quantification of retinal microvascular density in optical coherence tomographic angiography images in diabetic retinopathy. JAMA Ophthalmol 2017; 1;135(4): 370–37. 10.1001/jamaophthalmol.2017.0080 28301651 PMC5470403

[9] Palejwala NV, Jia Y, Gao SS, Liu L, Flaxel CJ, Hwang TS et al Detection of non-exudative choroidal neovascularization in age-related macular degeneration with optical coherence tomography angiography. Retina 2015; 35: 2204–2211. 10.1097/IAE.0000000000000867 26469533 PMC4623999

[10] Gołębiewska J, Brydak-Godowska J, Moneta-Wielgoś J, Turczyńska M, Kęcik D, Hautz W. Correlation between choroidal neovascularization shown by OCT Angiography and choroidal thickness in patients with Chronic Central Serous Chorioretinopathy. J Ophthalmol Article ID 3048013. 10.1155/2017/3048013 PMC5646334 29109866

[11] Fan H, Chen HY, Ma HJ, Chang Z, Yin HQ, Ng DS et al Reduced macular vascular density in myopic eyes. Chin Med 2 2017;130(4): 445–451. 10.4103/0366-6999.199844 PMC5324382 28218219

[12] Li M, Yang Y, Jiang H, Gregori G, Roisman L, Zheng F et al Retinal microvascular network and microcirculation assessments in high myopia. Am J Ophthalmol 2017; 2;174:56–67. 10.1016/j.ajo.2016.10.018 27818204 PMC5253241

[13] Al-Sheikh M, Phasukkijwatana N, Dolz-Marco R, Rahimi M, Iafe NA, Freund KB et al Quantitative OCT Angiography of the retinal microvasculature and the choriocapillaris in Myopic Eyes. Invest Ophthalmol Vis Sci 2017; 4 1;58(4):2063–2069. 10.1167/iovs.16-21289 28388703

[14] Mo J, Duan A, Chan S, Wang X, Wei W. Vascular flow density in pathological myopia: an optical coherence tomography angiography study. BMJ Open 7: e013571 10.1136/bmjopen-2016-013571 28159853 PMC5294002

[15] Linderman R, Salmon AE, Strampe M, Russillo M, Khan J, Carroll J. Assessing the accuracy of foveal avascular zone measurements using optical coherence tomography angiography: segmentation and scaling. Trans Vis Sci Tech. 2017;6(3):16. 10.1167/tvst.6.3.16 PMC5469394 28616362

[16] Sampson DM, Gong P, An D, Menghini M, Hansen A, Mackey DA, et al Axial length variation impacts on superficial retinal vessel density and foveal avascular zone area measurements using optical coherence tomography angiography. Invest Ophthalmol Vis Sci. 2017;58:3065–3072. 10.1167/iovs.17-21551 28622398

[17] Dhami A, Dhasmana R, Nagpal RC. Correlation of Retinal Nerve Fiber Layer thickness and axial length on Fourier Domain Optical Coherence Tomography. J Clin Diagn Res 2016; 4, Vol-10 (4): NC15–NC17. 10.7860/JCDR/2016/15038.7672 PMC4866148 27190850

[18] Sezgin Akcay BI, Gunay BO, Kardes E, Unlu C, Ergin A. Evaluation of the Ganglion Cell Complex and Retinal Nerve Fiber Layer in low, moderate, and high myopia: A Study by RTVue Spectral Domain Optical Coherence Tomography. Semin Ophthalmol 2016; 7 12:1–7. 10.3109/08820538.2016.1170157 27404600

[19] Maruko I, Iida T, Sugano Y, Oyamada H, Akiba M, Sekiryu T. Morphologic analysis in pathologic myopia using high-penetration Optical Coherence Tomography. Invest Ophthalmol Vis Sci 2012; 53:3834–3838. 10.1167/iovs.12-9811 22589433

[20] Shimada N, Ohno-Matsui K, Harino S, Yoshida T, Yasuzumi K, Kojima A et al Reduction of retinal blood flow in high myopia. Graefes Arch Clin Exp Ophthalmol 2004: 242:284 10.1007/s00417-003-0836-0 14722781

[21] Zheng Q, Zong Y, Li L, Huang X, Lin L, Yang W et al Retinal vessel oxygen saturation and vessel diameter in high myopia. Ophthalmic Physiol Opt 2015; 35:562–569. 10.1111/opo.12223 26303449

[22] Gupta P, Thakku SG, Saw SM, Tan M, Lim E, Tan M et al Characterization of choroidal morphologic and vascular features in young men with high myopia using Spectral-Domain Optical Coherence Tomography. Am J Ophthalmol 2017; 5 177:27–33. 10.1016/j.ajo.2017.02.001 28209502

[23] Hirata A, Negi A. Morphological changes of choriocapillaris in experimentally induced chick myopia. Graefes Arch Clin Exp Ophthalmol 1998; 236(2):132–137. 9498124 10.1007/s004170050053

[24] Yang YS, Koh JW. Choroidal blood flow change in eyes with high myopia. Korean J Ophthalmol 2015; 29:309–314. 10.3341/kjo.2015.29.5.309 26457036 PMC4595256

